# Politicians’ email communication with voters: A field experiment for the 2021 Bundestag elections

**DOI:** 10.1371/journal.pone.0324542

**Published:** 2025-07-22

**Authors:** Ekkehard A. Köhler, Marius D. May

**Affiliations:** University of Siegen, Siegen, Germany; Universität Hamburg: Universitat Hamburg, GERMANY

## Abstract

First-time voters hold polarized views on migrant issues. Few is known about how politicians react to this peculiar phenomenon in democracies. This paper studies whether rational behavior drives political elites’ response behavior in a RCT email responsiveness study. We contacted 1554 candidates for the German Bundestag shortly before the 2021 election and asked about their stance on dual citizenship. We went into the field as first-time voting high-school students and varied their stances on dual citizenship, migration backgrounds and genders. Introducing a theoretical framework based on the single-policy task model and supplementing it with costly persuasion, we are able to disentangle and test for partisanship and opportunism. Our main results are as follows: First, both partisanship and opportunism explain the candidates’ response behavior. Second, candidates exert more effort if they disagree with a voter than if they agree with a voter.

## 1 Introduction

Legislators’ response behavior to their constituents has been subject to a large body of experimental studies [1]. However, less is known about the prevalence of vote-maximizing motives from political economy in such experiments. This is true even though partisanship (e.g. [2–4]) and opportunism (e.g. [5–7]) are well-researched phenomena in theoretical and empirical political economy.

We investigate whether these facets of rational behavior also prevail in email communication between voters and candidates during campaigning before an election. In addition, we shed light on the question whether minority voters are discriminated against if the underlying issue of the inquiry is both controversial and related to migration. To do so, we develop a model of the candidate’s decision making that is inspired by the single-policy task model by [8] and theoretical literature on costly persuasion (e.g. [9–13]). The experimental design is as follows: Candidates for the German Bundestag receive an inquiry from a female or male first-time voter with or without migration backgrounds. Each message conveys some alignment with the candidate, an information signal on the voter’s (un-)critical stance on dual citizenship and ends with a question if the candidate advocates dual citizenship.

Our analyses yields the following main results: Disentangling partisanship and opportunism in the experimental design, we find evidence of both in the candidates’ communication patterns. Moreover, we find that candidates tend to make a higher effort if they disagree with a voter than if they agree with a voter. We find limited evidence of discrimination against minority voters. In addition, we do not find discrimination against voters with migration backgrounds in terms of lower response rates. Finally, we neither find that female voters receive a response at a lower chance.

Our study makes significant contributions to the corresponding literature in several ways.

First, as outlined above, to the best of our knowledge, we are the first to investigate and disentangle partisanship and opportunism at the same time in an audit study with political elites. Our results suggest that such facets of rational behavior should be carefully considered in the design stage of such field experiments. Furthermore, analyzing the content of replies in addition to an analysis of response rates, can reveal a richer and more nuanced set of results.

Second, we introduce not only a policy-oriented question in our audit study (as, e.g., [14,15]), but also inquirers with a polarized stance on this issue. This additional feature provides valuable insights and affects the politicians’ responsiveness.

Third, our results suggest that issues related to minority voters, such as dual citizenship, may diminish discrimination against minority voters (see especially [1]). Further audit studies with political elites should take this insight into account at the design stage. More research may further elucidate the relationship between discrimination against minority voters and the issue of inquiries.

Fourth, our results add to the growing body of literature on the behavior of political elites towards different voter types (e.g. [16–18]). We show that distinguishing between voters who are aligned or unaligned with a candidate’s party is key to understand the response behavior precisely. We do not find any evidence to conclude discriminatory behavior against women or against voters with Turkish migration background.

Fifth, we establish a model that allows us to motivate different hypotheses on rational behavior by candidates within our experimental design. This model may serve as a starting point for various further research questions related to communication between voters and political elites. Thus, the framework can help answer more questions about rational behavior of candidates than those featured in our study.

The remainder of this paper is structured as follows: Sect 2 provides a theoretical model that defines the expected behavior in the correspondence study. Sect 3 provides an overview of the experimental design, methods and the institutional context of the experiment with a special focus on the varying stances towards dual citizenship between parties. Based on the theoretical framework and the peculiar context in the German Federal election campaign, we formulate three hypotheses in Sect 4. Results are presented in in numerical order in Sect 5. We discuss these results and conclude our study with further questions in Sect 6.

## 2 Theoretical framework

In the following section, we develop a theoretical framework to derive the hypotheses on rational behavior by candidates. The model builds on the single-policy task model by [8] and on the modeling of costly persuasion in information economics (e.g. [9–12,19]). We consider our experiment as a sender-receiver game where a first-time voter asks for the candidate’s stance on dual citizenship. Moreover, the voter signals in the email some general alignment with the candidate. The candidate can attempt to further align the first-time voter by sending a reply. The candidate then takes the role of the sender and the voter the role of the receiver. The candidate’s decision tree is now as follows: First, the candidate has to decide whether to answer or not. Second, if the candidate decides to answer, the candidate can agree or disagree with the inquirer. Finally, the candidate chooses an effort level, which we consider binary as low or high for the sake of simplicity.

The candidate’s decision tree therefore looks as follows:



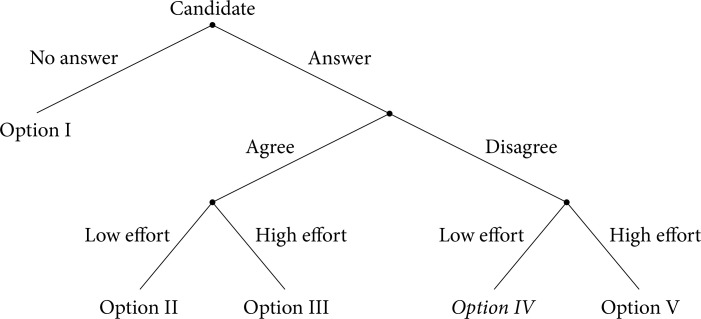



The candidate’s payoff *u* consists of the following components: a reward *R*, communication costs *c* and lying costs *Y*, with u(R,c,Y)=R−c−Y. This composition builds on the model by [8], models of costly communication by senders (e.g. [9,12]) and persuasion models involving lying costs (e.g. [10,13,20]). We elaborate on each component in the following.

The reward *R* depends on the candidate’s success in convincing the voter of the candidate’s stance on dual citizenship. If the voter is not convinced, *R* = *L*<0 and *R* = *H*>0 if the voter is convinced. Additionally, *R* = 0 if the candidate does not respond at all. This definition highlights an incentive for the candidate to persuade the inquirer to maximize the chances of being elected. However, an answer may deteriorate these chances if the candidate fails to convince the voter of the candidate’s stance with an answer. This can only happen if the candidate disagrees with the inquirer. If the candidate disagrees with the inquirer, the probability of convincing the voter and gaining the reward *R* = *H* is *p*_*h*_ making a high effort and *p*_*l*_ making a low effort, with $0<p_{l}<p_{h}<1$. The difference is Δp, with $\Delta p=p_{h}\;-\;p_{l}$. The probabilities that the voter is misaligned, leading to reward *R* = *L* are each the complementary probability $1\;-\;p_{h}$ and $1\;-\;p_{l}$, respectively. This assumption is in line with theoretical literature on costly and Bayesian persuasion (e.g. [9,11,12,21,22]). The more effort a sender makes, the greater is the chance of convincing a receiver who updates beliefs accordingly. For simplicity, we further assume that the candidates are risk neutral. Furthermore, we assume that agreeing with the inquirer always yields a high reward *H* even if the candidate lies in doing so. This means that candidates do not only lie, but also deceive voters successfully (see especially [19]).

Communication costs *c* depend on the level of effort chosen by the candidate. If the candidate decides not to answer, *c* = 0. Low effort incurs *c* = *e* and high effort incurs *c* = *E*, with 0<*e*<*E*. In addition, Δe=E−e. Intuitively, exerting more effort means a more time-consuming answer. A low-effort answer might include just a simple statement of agreement or disagreement while a high-effort answer might include an elaborate portfolio of arguments supporting the candidate’s stance. We do not make a specific assumption about how cost levels *e* and *E* differ. Some literature, e.g. [8,9], suggests that costs increase disproportionately in effort. Other work assumes less restrictive assumptions on the sender’s communication costs and also allow for lower increases in effort (e.g. [11–13,22]). Intuitively, the latter strand of literature suits our experiment better because there may be intrinsically motivated candidates or candidates who have well-prepared arguments at hand (e.g. from manifestos) so that high efforts do not incur disproportionate costs. Finally, we do not take into account the potential communication costs of the inquirer. This is mainly due to two reasons. First, an inquirer signals a genuine interest in understanding the candidate’s stance. This makes reading a reply less exhausting. Second, receiving a high-quality response can be pleasant because a voter feels catered to.

Finally, the lying costs are *Y* = *y*>0 if the candidate’s revealed stance on dual citizenship in the answer does not concur with the candidate’s party’s stance and *Y* = 0 otherwise. Thus, for the sake of simplicity, we assume that the candidate’s stance on dual citizenship is always aligned with the stance on dual citizenship represented by the candidate’s party. Lying costs have first been introduced to sender-receiver games by [10,20]. In our experiment, lying costs can occur due to the intrinsic aversion to lying (see, e.g., [23–25] for experimental evidence) and additional effort to falsify a stance by, e.g., finding arguments contrary to the true stance. [13] provides a distinction between different types of lying costs.

With these definitions of the utility function and its components at hand, we can formulate the expected utilities for each option of the decision tree. Notice that a distinction between voters aligned and not aligned with the candidate’s party is needed here. The expected utilities are as in Table 1.

**Table 1:** Candidate’s expected utilities.

	Voter aligned with party stance (A)		Voter unaligned with party stance (NA)	
**Candidate’s decision**	**Option**	***u*(*R*,*c*,*Y*)**	**Option**	***u*(*R*,*c*,*Y*)**
No answer	A.I	0	NA.I	0
Agree; low effort	A.II	*H*–*e*	NA.II	*H*–*e*–*y*
Agree; high effort	A.III	*H*–*E*	NA.III	*H*–*E*–*y*
Disagree; low effort	A.IV	$p_{l}H+(1-p_{l})L-e-y$	NA.IV	$p_{l}H+(1-p_{l})L-e$
Disagree; high effort	A.V	$p_{h}H+(1-p_{h})L-E-y$	NA.V	$p_{h}H+(1-p_{h})L-E$

Notice that for a candidate facing a voter aligned with the party’s stance on dual citizenship, agreeing with a low effort (A.II) is the best option of those which features an answer at all. This is not true for candidates confronted with a unaligned voter because agreeing involves lying costs *y*. Additionally, agreeing each with low- and high-effort is more profitable for candidates facing an aligned voter than for candidates facing an unaligned voter. The opposite is true for disagreeing. Finally, agreeing with an unaligned voter choosing high effort (NA.III) is strictly inferior to agreeing with an unaligned voter choosing low effort (NA.II) because of communication costs and the assumption that agreeing always convinces the inquirer.

We will outline the stances on dual citizenship represented by different parties in the next section. This allows us to define what voters are aligned and unaligned in terms of our theoretical framework.

## 3 Data and methods

We adopt the approach of a correspondence study [26,27]. In this study, we used a treatment design of 2 * 2 * 2. The first treatment dimension focused on the inquirer’s binary perspective on dual citizenship, with inquirers either skeptical about dual citizenship or not. More details on the institutional and political context of dual citizenship will be discussed later in this section. The second treatment revolved around the inquirer’s race, using first names derived from German census data to signal either Turkish migration backgrounds or none. Notice that choosing names signaling a Turkish descent is a very viable option to examine discrimination against minorities in Germany (see, e.g., [15]). Surnames did not convey any information about migration backgrounds in this study. First names are an adequate indicator of whether or not an arbitrary person has migration backgrounds in Germany. We wanted to increase external validity with this choice: A person with a Turkish first name but a German last name is very likely to have a German father and a Turkish mother. The issue of dual citizenship is very relevant to the children of these couples. Note that according to German law, you may choose the birth or current name of either spouse as a married couple. However, around 90 percent of couples chose the name of the husband as the family name. The third treatment relates to gender, indicating whether the inquirer was female or male. Combining the first treatment with the other two, we used four distinct names: Lena Müller, Azra Müller, Yusuf Wolf, and Linus Wolf.

It is important to note that the first treatment pertained to the inquirer’s ideology, while the second and third treatments varied the inquirer’s sociodemographic characteristics. This allowed us to investigate what drives candidate responsiveness. Specifically, we sought to determine whether candidates aimed to maximize votes by considering the inquirer’s stance on dual citizenship and whether candidates exhibited biases against particular voter types. This multifaceted design is more comprehensive and distinct compared to studies that focus on a single characteristic, such as race (e.g. [28–30]).

Although incorporating a policy issue into studies is not new (e.g. [14]), we introduced inquirers who had polarized views on the issue as a novel feature. Although other studies differentiate between issue-related and service-related questions to investigate the impact of request nature on response behavior, we were interested in understanding how candidates handle polarized voter inquiries in this experiment. In addition, in the emails we conveyed that the inquirers were first-time voters who attended high school. This is another unique aspect of our study. First-time voters represent a significant voting potential for politicians and have polarized views on migration issues.

The following text is a translation from the text we used in our emails:

Subject: Request from first-time voter

Dear [Mrs./Mr.] [surname],

my name is [Lena Müller/Azra Müller/Linus Wolf/Yusuf Wolf] and I could imagine voting for you on September 26.

As part of our school lessons, I was confronted with the topic of dual citizenship, which I view [un]critically.

Do you advocate dual citizenship for citizens with migration backgrounds?

Thank you for your reply!

Kind regards,

[Lena Müller/Azra Müller/Linus Wolf/Yusuf Wolf]

Our dataset comprises 1554 candidates from the six parties represented at that time in the German Bundestag. These parties include the Left party, the Greens, the Social Democratic Party (SPD), the Free Democratic Party (FDP), the Christian Democratic Union/Christian Social Union (CDU/CSU) and the Alternative for Germany (AfD). We also included candidates from the "Free voters" (FW) party in Bavaria, where the party is part of the government coalition. We collected the email addresses between September 1, 2021 and September 5, 2021. Candidates without valid email addresses were excluded from our contact list. We applied randomization of the 2*2*2 treatments across all candidates at the party level to explore differences in responsiveness among candidates from different parties.

Our dataset encompasses various types of control data, including candidate-specific variables (e.g., age, party, gender, incumbency and migration backgrounds as a binary variable) and district-specific variables (e.g., the proportion of foreigners, household income and voter turnout in 2017).

We sent the emails automatically on September 16 and September 17, 2021. The Candidates were not informed that they participated in an experiment. This is a common procedure for such field experiments. If an email was blocked or failed to send successfully, we removed the respective candidate from our sample. We tracked whether we received manually typed responses or not until September 26, 2021, which marked the day of the election. The binary observation was assigned a value of 1 for a manually typed response and 0 otherwise. We achieved a response rate of 67.57%. This rate is relatively high compared to related studies on political elites (as indicated by the meta-study by [1]) and to audit studies on other forms of discrimination (e.g. [31–35]). This high response rate suggests that our messages were taken seriously. Furthermore, it implies that candidates prioritize responding to the needs of students and first-time voters. Finally, it indicates that dual citizenship was a relevant issue to include in our study.

In addition, answers covering a stance on dual citizenship were also classified. There is a category each for responses stating a clear preference in favor or against dual citizenship. These answers are especially relevant. Other categories comprise replies showing stances that do not fit this polarized distinction. In the results section, we report t-tests on the mean differences in line with the underlying hypotheses, with the corresponding figures displaying 90% confidence intervals.

Moreover, we measured the length of responses. The response length serves as a proxy for the candidate’s effort. Email addresses, closing remarks, and quotes from the election program were excluded from consideration. This approach allowed us to focus on the manually written content within the responses and obtain a more precise measure of effort.

German citizenship is granted according to the principle of decent (ius sanguinis) such that a child acquires German citizenship at birth if one of his or her parents is a German citizen. The idea of dual citizenship is to ease the path to citizenship for non-Germans living and working in the federal republic such that they become a "naturalized" citizen without giving up the previous citizenship. Dual citizenship was facilitated by federal legislation in August 2023. During the federal election in 2021, dual citizenship was a controversial and polarized issue in the election campaign between left and right parties. For millions of young people in Germany, new regulations could improve participation in the labor market.

Importantly for our study, parties featured different stances on dual citizenship during the campaigning phase for the federal elections in 2021. The Left party, the Greens and the SPD supported dual citizenship without restrictions. In contrast, the AfD opposed dual citizenship. In terms of our theoretical framework in Sect 2, aligned and unaligned voters are as follows: Voters signaling support of dual citizenship are aligned with the Left party, the Greens and the SPD and unaligned with the AfD. Further, voters signaling opposition to dual citizenship are aligned with the AfD and unaligned with the Left party, the Greens and the SPD. The stances on dual citizenship represented by the FDP, CDU/CSU and FW did not fit in this polarized pattern. Their policy suggestions featured dual citizenship with some restrictions, but not a clear opposition.

Ethical concerns are negligible in our experiment. It received IRB approval and is designed in accordance with the guidelines established by [36]. We investigate the motives making legislators respond to voters’ requests, which is an important question. In this regard, we build on similar field experiments and extend the setting with a policy-related question. The latter is subject of other studies as mentioned above (e.g., [14]). We gathered both individual-level data and context-level data to maximize the validity of the experiment. Second, in line with [36], we minimized collective costs for political elites. The time it takes to answer the question included in the email is very short. This is true since only an opinion and not information is required and parties provided their stances on dual citizenship in manifestos. We also indicated unambiguously that the stance represented in the email was only the voter’s individual stance. This minimizes information deception.

## 4 Hypotheses

In the following section, we formulate and elucidate our hypotheses. We focus on hypotheses motivated by the theoretical framework developed in Sect 2. Our analyses show that both the sender’s migration background and the sender’s gender are irrelevant for response rates. Thus, we restrict ourselves to the analysis of the hypotheses motivated by the theoretical model. In a nutshell, our hypotheses are motivated as follows: Candidates hold ideological views on dual citizenship (partisan motive) but want to maximize votes at the same time (vote maximization or opportunistic motive). Replying to a mail is costly. Not replying to a mail can result in the loss of a supporter. With enough effort, one may hope to convince a voter who does not share the politician’s views.

Our first hypothesis addresses partisanship effects, which suggest that certain parties are more responsive to voters who share their party’s stance on a particular policy. We expect partisanship effects in a sense that response rates towards voters aligned with the party’s stance on dual citizenship (A) are higher than to unaligned voters (NA). This hypothesis stems from our model: In order to make a candidate answer to an aligned voter, the utility from answering choosing a low effort (A.II) has to be more profitable than not answering at all (A.I). This is true iff:


e<H

For candidates facing an unaligned voter (NA), at least one of the options NA.II, NA.IV and NA.V has to be superior to not answering (NA.I).

*e* + *y*<*H* for (NA.II)

$e<p_{l}H+(1-p_{l})L$ for (NA.IV)

$E<p_{h}H+(1-p_{h})L$ for (NA.V)

Notice that each condition is more restrictive than the condition to make A.II superior to A.I.. Thus, we expect a higher response rate towards aligned voters than to unaligned voters. Therefore, we formulate the following hypothesis on partisanship effects:

**Hypothesis 1** (Partisanship Effects) *Politicians are more likely to reply to voters whose position on dual citizenship is aligned with their own than to unaligned voters.*

Next, we anticipate that opportunism influences the stance a candidate conveys in their response. We identify opportunism as follows: Candidates are more likely to reveal an arbitrary stance on dual citizenship if it matches the inquirer’s stance than if it does not match the inquirer’s stance. According to our model, this requires a candidate facing a voter aligned with the party’s stance (A) to agree (A.II and A.III). As discussed, agreeing with low effort to an aligned voter is superior to all other options including an answer. Moreover, candidates facing an unaligned voter (NA) also need to agree. For agreeing with an unaligned voter choosing low effort (NA.II) to be more beneficial than disagreeing with the voter, both of the following conditions have to be true:

*y*<(1−*p*_*l*_)(*H*−*L*) for (NA.IV)

$y-\Delta c<(1-p_{h})(H-L)$ for (NA.V)

The conditions show that a candidate rather tends to persuade a voter than to both lie to and agree with a unaligned voter if lying costs are high and the low reward is close to 0. Additionally, if choosing an high effort involves relatively low additional costs and a relatively high chance to gain a high reward compared to choosing a low effort, the candidate tends to opt for disagreeing with a high effort. We formulate the hypothesis on opportunism as follows:

**Hypothesis 2 (Opportunism).** *Opportunistic behavior by candidates is expected as follows.*

- *The candidate’s answer signals a stance in favor of dual citizenship at a higher chance if the inquirer favors dual citizenship than if the inquirer opposes dual citizenship.*
- *The candidate’s answer signals a stance against dual citizenship at a higher chance if the inquirer opposes dual citizenship than if the inquirer supports dual citizenship.*

Notice that it would also be possible to simplify hypothesis 2 in a sense that a distinction between support of and opposition to dual citizenship revealed in the candidate’s answer is left out. Yet, this simplified hypothesis would not be testable with a t-test but only with a mean comparison. This is why we stick to the distinction in hypothesis 2.

Additionally, we study whether candidates spend more effort if they disagree with a voter than if they agree with a voter. We use the number of words in the responses as a measure. The intuition behind this hypothesis is that candidates may need more effort in order to convince a voter who signals a stance that is contrary to the candidate’s stance. This hypothesis requires that choosing a high effort is more beneficial than a low effort for a disagreeing answer and vice versa for agreeing answers. In other words: A.II has to be superior to A.III, A.V has to be superior to A.IV, NA. II has to be superior to NA.III and NA.V has to be superior to NA.IV. The first condition is true, the second condition is obsolete as A.II is superior to A.IV and A.V. The third condition is also true. The fourth condition, i.e., disagreeing with an unaligned voter is more profitable with a high effort than with a low effort, is met iff:

$$\Delta_{p}(H-L)>\Delta_{c}$$

If the increased chance to gain a high reward by successfully convincing the voter excels the additional costs due to a high effort, a candidate will choose a high level for disagreement with a unaligned voter. We formulate the following hypothesis:

**Hypothesis 3 (Adaptation of effort level).** *Conditional on replying, candidates spend more words when replying to voters with whom they disagree.*

## 5 Results

Summary statistics (Table 2 in the Appendix) and robustness checks (Tables 3, 4, 5, 6, 7, 8, 9, and 10 in the Appendix) for the results can be found in the appendix.

**Table 2:** Summary statistics.

Variable	N	Mean	Std. dev.	Min.	Max.
Reply rate	1554	0.676	0.468	0	1
Mean reply length	1554	130.52	105.42	2	843
Left Party	1554	0.163	0.370	0	1
Greens	1554	0.163	0.369	0	1
SPD	1554	0.163	0.370	0	1
FDP	1554	0.161	0.367	0	1
CDU/CSU	1554	0.183	0.387	0	1
FW	1554	0.029	0.168	0	1
AfD	1554	0.138	0.345	0	1
Female candidate	1554	0.308	0.462	0	1
Age candidate	1554	46.288	12.557	18	80
Candidate with migration backgrounds	1554	0.058	0.234	0	1
GDP per capita 2018	1554	39695.36	14685.063	21403	110283
Unemployment rate in %	1554	6.307	2.311	2.8	15.8
Inhabitants per square km	1554	849.099	1153.558	36.81	4777.04
Share of people with migration backgrounds in%	1554	25.442	9.210	6.67	38.78
Voter turnout in 2017 in %	1554	76.19	3.36	64.73	83.88

**Table 3:** T-test on the result of Hypothesis 1.

Voter unaligned with candidate’s party	
N	474
Mean reply rate	61.39%
Voter aligned with candidate’s party	
N	501
Mean reply rate	75.05%
Differential	-13.66
$H_{a}:differential\neq 0$	*p* = 0.00
*H*_*a*_:*differential*<0	*p* = 0.00
*H*_*a*_:*differential*>0	*p* = 1.00

**Table 4:** Partisanship effects on side of parties supporting dual citizenship.

	Left Party	Greens	SPD
Inquirer opposes dual citizenship			
N	123	121	122
Mean reply rate	77.24%	62.81%	61.48%
Inquirer supports dual citizenship			
N	131	132	132
Mean reply rate	74.05%	83.33%	78.79%
Differential	3.19	-20.52	-17.31
$H_{a}:differential\neq 0$	*p* = 0.56	*p* = 0.00	*p* = 0.00
*H*_*a*_:*differential*<0	*p* = 0.72	*p* = 0.00	*p* = 0.00
*H*_*a*_:*differential*>0	*p* = 0.28	*p* = 1.00	*p* = 1.00

**Table 5:** Partisanship effects on side of parties opposing dual citizenship.

	AfD
Inquirer opposes dual citizenship	
N	106
Mean reply rate	61.32%
Inquirer supports dual citizenship	
N	108
Mean reply rate	41.67%
Differential	19.65
$H_{a}:differential\neq 0$	*p* = 0.00
*H*_*a*_:*differential*<0	*p* = 1.00
*H*_*a*_:*differential*>0	*p* = 0.00

**Table 6:** T-test on the result of Hypothesis 2a.

Inquirer opposes dual citizenship	
N	517
Candidate signals support of dual citizenship	43.32%
Inquirer supports dual citizenship.	
N	581
Candidate signals support of dual citizenship	62.31%
Differential	-18.98
$H_{a}:differential\neq 0$	*p* = 0.00
*H*_*a*_:*differential*<0	*p* = 0.00
*H*_*a*_:*differential*>0	*p* = 1.00

**Fig 1:**
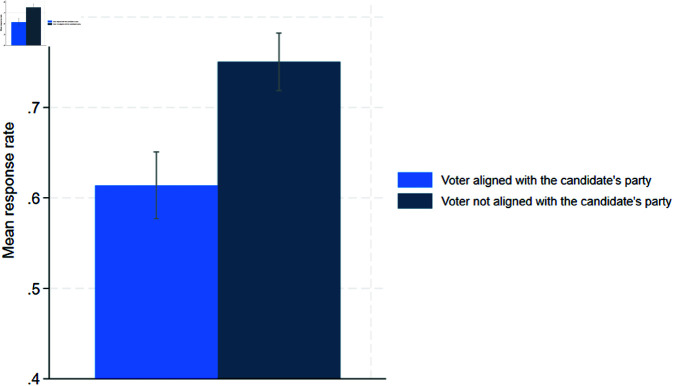
Hypothesis 1.

First, we examine Hypothesis 1, which covers the partisanship effects:

Fig 1 illustrates significant partisanship effects. Voters aligned with the candidate’s party receive an answer with a higher chance than voters that are not aligned with the candidate’s party (75.05% and 61.39%, respectively). Based on this evidence, we confirm Hypothesis 1. To substantiate the robustness of our results, we performed a t-test (Table 3 in the Appendix). This test shows significant partisanship effects at the 99% level.

We also tested for partisanship effects on the party level. At this, we expect that candidates of the Left Party, the Greens and the SPD respond to a voter who supports dual citizenship at a higher chance than to a voter who opposes dual citizenship. Likewise, we expect that candidates of the AfD respond to a voter who opposes dual citizenship at a higher chance than to a voter who supports dual citizenship. The results can be summarized as follows (see Tables 4 and 5 in the Appendix): Supporters of dual citizenship have a significantly higher chance of receiving a response from the Greens and Social Democrats (83.33% and 78.79%, respectively) than opponents of dual citizenship (62.81% and 61.48%, respectively). These partisanship effects are significant at the 99% level. Moreover, opponents of dual citizenship receive a response from AfD candidates at a significantly higher probability than supporters of dual citizenship (61.32% vs. 41.67%). This effect is also significant at the 99% level. However, contrary to our expectations, we do not find such a difference for candidates of the Left party. The chances of receiving an answer from a candidate from the Left party are 77.24% for opponents of dual citizenship and 74.05% for supporters of dual citizenship.

**Fig 2:**
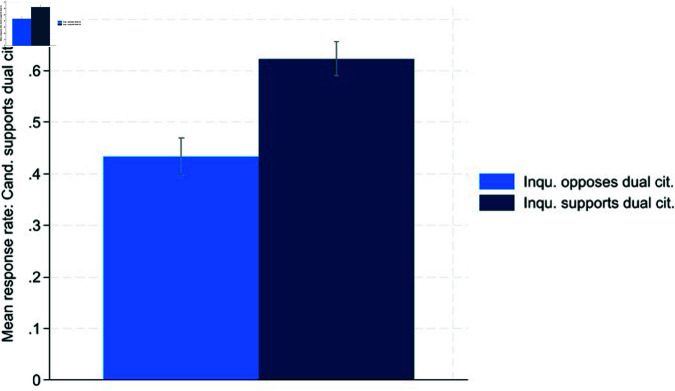
Pro dual citizenship.

**Fig 3:**
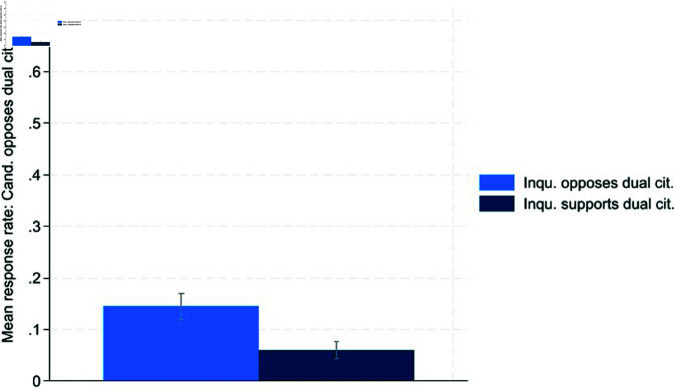
Contra dual citizenship.

Next, we investigate opportunistic behavior by candidates as outlined in Hypotheses 2a and 2b. The results are depicted in Figs 2 and 3:

Figs 2 and 3 provide evidence of opportunistic behavior as defined in Hypothesis 2. When facing a supporter of dual citizenship, candidates signal significantly more often that they also favor dual citizenship (62.31%) than if the inquirer is against it (42.31%). Additionally, inquirers opposing dual citizenship receive an answer revealing an opposing stance by the candidate significantly more often than inquirers conveying a supporting stance (14.53% vs. 6.02%). This evidence supports the conclusion that both parts of Hypothesis 2 hold. T-tests confirm that opportunistic behavior is significant at the 99% level (Tables 6 and 7 in the Appendix). We also performed logistic regressions with various control variables to examine what determines a candidate’s odds of favoring or opposing dual citizenship (Tables 8 and 9 in the Appendix). These robustness checks provide additional evidence of opportunistic behavior, as the inquirer’s opinion on dual citizenship is significant at the 99% level.

**Table 7:** T-test on the result of Hypothesis 2b.

Inquirer opposes dual citizenship	
N	517
Candidate signals opposition to citizenship	14.51%
Inquirer supports dual citizenship.	
N	581
Candidate signals opposition dual citizenship	6.02%
Differential	8.48
$H_{a}:differential\neq 0$	*p* = 0.00
*H*_*a*_:*differential*<0	*p* = 1.00
*H*_*a*_:*differential*>0	*p* = 0.00

**Table 8:** Logit estimation on Hypothesis 2a.

	(1)	(2)
**Variable**	**Logits**	**Margins**
Inquirer supports dual citizenship	2.25***	0.509***
	(0.310)	(0.0574)
Inquirer with migration background	0.389*	0.0963*
	(0.232)	(0.0569)
Female inquirer	0.264	0.0657
	(0.228)	(0.0566)
Dummy AfD	–8.27***	–0.731***
	(0.980)	(0.0253)
Dummy FW	–5.48***	–0.555***
	(0.910)	(0.0362)
Dummy CDU/CSU	–6.05***	–0.772***
	(0.520)	(0.0318)
Dummy FDP	-5.08***	-0.720***
	(0.462)	(0.0373)
Dummy SPD	-0.508	-0.126
	(0.415)	(0.102)
Dummy Greens	-0.218	-0.0543
	(0.431)	(0.108)
Dummy female cand.	0.124	0.0307
	(0.247)	(0.0611)
Age cand.	-0.034***	-0.00849***
	(0.0097)	(0.00241)
Dummy cand. incumbent	0.014	0.00354
	(0.388)	(0.0965)
Share of women in %	18.08	4.50
	(20.45)	(5.09)
Inhabitants per square km	-0.000209	-0.000052
	(0.00022)	(0.00005)
Share of foreigners in %	11.88**	2.96**
	(5.19)	(1.29)
Voter turnout in 2017 in %	5.46	1.36
	(4.87)	(1.21)
Household income in	-0.000165**	-0.000041**
	(0.000082)	(0.00002)
GDP per capita in	–6.47*10^−6^	–1.61*10^−6^
	(0.000015)	(0.00000)
Unemployment rate in %	0.0366	0.00911
	(0.116)	(0.0288)
Constant	-7.45	
	(9.59)	
Observations	1,098	1,098
Robust standard errors in parentheses; Pseudo R2: 0.646		
*** p<0.01, ** p<0.05, * p<0.1		

**Table 9:** Logit estimation on Hypothesis 2b.

	(1)	(2)
**Variable**	**Logits**	**Margins**
Inquirer supports dual citizenship	-1.52***	-0.0495***
	(0.346)	(0.0185)
Inquirer with migration background	-0.0868	–0.00260*
	(0.309)	(0.00928)
Female inquirer	-0.114	-0.00341
	(0.314)	(0.00961)
Dummy AfD	5.76***	0.791***
	(0.747)	(0.0859)
Dummy FW	1.57	0.0985***
	(1.30)	(0.140)
Dummy CDU/CSU	3.50***	0.271***
	(0.728)	(0.0972)
Dummy FDP	-0.693	-0.0182
	(1.22)	(0.0266)
Dummy female cand.	-0.627	-0.0163
	(0.390)	(0.0107)
Age cand.	0.0111	0.000334
	(0.0145)	(0.00045)
Dummy cand. incumbent	-1.52***	-0.0317**
	(0.518)	(0.0125)
Share of women in %	-7.56	-0.227
	(28.16)	(0.855)
Inhabitants per square km	0.00022	6.60*10^−6^
	(0.00029)	(0.00001)
Share of foreigners in %	–10.68*	–0.321*
	(5.62)	(0.180)
Voter turnout in 2017 in %	-0.701	-0.0211
	(5.83)	(0.175)
Household income in	-0.000133	–4.00*10^−6^
	(0.000118)	(0.00000)
GDP per capita in	0.000022*	6.66*10^−7^
	(0.000013)	(0.00000)
Unemployment rate in %	-0.116	0.00350
	(0.161)	(0.00481)
Constant	3.87	
	(13.63)	
Observations	723	723
Robust standard errors in parentheses; Pseudo R2: 0.535		
*** p<0.01, ** p<0.05, * p<0.1		

**Fig 4:**
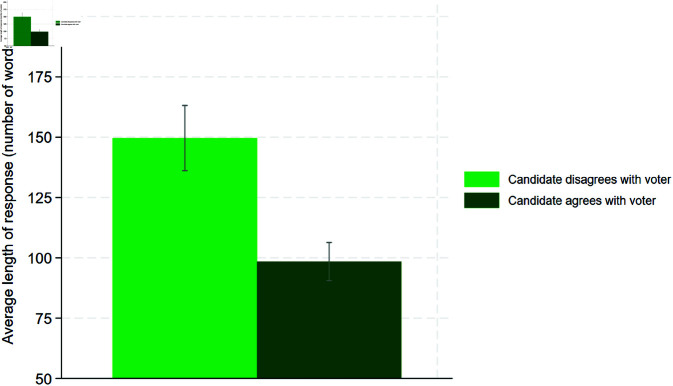
Hypothesis 3.

As a next step, we test Hypothesis 3 on the candidate’s effort. The results are as follows:

Fig 4 illustrates that candidates who agree with the voter write longer responses than candidates who disagree with the voter (149.63 words vs. 98.43 words). We can confirm Hypothesis 3 based on this evidence. A t-test supports this finding, indicating a significant difference in the mean response length at the 99% level (Table 10 in the Appendix).

**Table 10:** T-test on the results of Hypothesis 3.

Candidate disagrees with voter	
N	259
Average length of response	149.63%
Candidate disagrees with voter	
N	437
Average length of response	98.43%
Differential	51.19
$H_{a}:differential\neq 0$	*p* = 0.00
*H*_*a*_:*differential*<0	*p* = 1.00
*H*_*a*_:*differential*>0	*p* = 0.00

## 6 Discussion

Our study examines the response behavior of candidates for the German Bundestag in 2021 who face inquiries by first-time voters. Migration issues polarize young voters, especially in Germany. The experimental design allows us to investigate different facets of vote-maximizing behavior of candidates separately.

We have gathered substantial evidence to conclude that partisanship plays a significant role in our experiment. Therefore, this study underscores the importance of the literature on partisanship effects (e.g., [37–39]).

Our theoretical framework suggests why partisanship is utility-maximizing. Replying to unaligned voters either involves lying costs in case of agreement or both communication costs and a lack of a guarantee for successful persuasion in case of disagreement. These incentives not to reply to unaligned voters should be investigated in other settings, e.g., less polarized issues which feature lower lying costs.

One open question arising from the context of hypothesis 1 is why we do not observe any partisanship effects for candidates of the Left party. While we cannot definitively answer this question, we can offer one possible explanation for this result. The Left party was close to the five percent threshold required for parties to enter the German Bundestag based on secondary votes. This circumstance could have incentivized their candidates to serve any voter to increase their chances of exceeding the threshold. Intuitively, if parliamentary representation is at stake, it is better to serve anyone than only aligned voters. The fact that the Left party has the second-highest response rate among all parties considered (75.59%) supports this explanation. In terms of our model, the high reward *H* can be considered very high and the low reward *L* close to zero in this case.

As a new contribution to field experiments on the response behavior of political elites, we investigated whether candidates behave opportunistically. We found that candidates significantly more often agree than disagree with a polarized inquirer on the issue of dual citizenship. Importantly, this phenomenon holds true for both directions of polarization. There are three important take-aways from this analysis. First, a qualitative analysis of replies in audit study is a fruitful approach and leads to a more elaborate analysis than solely focusing on reply rates. Second, candidates show rational behavior in different ways: Partisanship is about whether the candidate replies and opportunism is about how the candidate replies in our study. Third, both our results and the framework motivating our hypotheses show that partisanship and opportunism, both highly relevant in political economy, can be disentangled. The framework shows that providing an answer at all is more attractive facing voters aligned to the party, suggesting partisanship. However, agreeing is often superior to disagreeing, even dealing with unaligned voters. Both higher lying costs and some probability that deception is not successful would lower the incentives to behave opportunistically, according to our model. Additional experiments can build on this hypothesis.

Our findings on opportunism are also relevant from a voter’s perspective. The voter’s stance significantly impacts the stance conveyed by the candidate. This is a concern because politicians should ideally base their responses on their own convictions rather than tailoring them to the inquirer’s opinion. Furthermore, this mechanism supports studies in information economics that investigate what information individuals convey in different situations (e.g. [40–42]). This insight raises the question of how candidates react on inquiries that do not signal any stance on the underlying issue. We expect that candidates, when faced with such an inquirer, would be more inclined to adhere to their party’s stance to persuade the inquirer without incurring lying costs.

Another novel feature in the literature is the examination of mean response length. In line with Hypothesis 3, we find longer responses by candidates who disagree with the inquirer. This supports the intuition that persuading a voter takes more effort than agreeing with a voter. According to our model, a candidate has an incentive to make a high effort if the expected benefits of an increased chance of convincing the voter outweigh higher communication costs. While we can confirm this theoretical implication, we cannot determine whether a low difference in communication costs or a high increase in the chance of concincing the voter are the main driver behind the result. Subsequent studies may attempt to disentangle these two drivers.

Similar to the results on opportunism, this aspect of the study also suggests that rational behavior in such experiments goes beyond merely measuring response rates. Our theoretical framework implies that exerting a high effort can be efficient in order to persuade an unaligned voter of the stance represented by the candidate’s party. Again, an experimental design which includes a voter without a specific stance can deepen this insight. Simple agreement is not possible in this case, so we would expect higher efforts from the candidates in order to persuade voters.

Comparing the results from partisanship effects and the variation of effort yields another insight. Partisanship implies that candidates tend to reply more often to inquirers who share their party’s stance on dual citizenship. Furthermore, candidates tend to use more words in their replies if they disagree with the inquirer. Combining these two aspects reveals that candidates aligned with their party are served more frequently, but unaligned voters receive more substantial responses due to the greater effort expended.

In conclusion, this study offers three key takeaways for future research. First, political elites can exhibit different facets of rational behavior in email communication with polarized voters. Second, it is possible to disentangle partisanship and opportunism, especially using both an analysis of response rates and of content. Third, political elites spend more communication effort if they disagree with voters than if they disagree with voters.

## Appendix

In the following, robustness checks and additional information on hypothesis 1 and hypothesis 2 are presented.

Notice that in the second logit estimation, observations from SPD and Green candidates are dropped due to collinearity. Candidates form the Left party are omitted in both logits for the same reason. Tables 8 and 9 show that the inquirer’s stance on dual citizenship affects the stance the candidate conveys in the response. The effects are each significant at the 99% level. Several party dummies also feature significant effects. This is as expected given the stances on dual citizenship the parties represent. It is also intuitive that inquirers with migration backgrounds and high shares of foreigners induce candidates to signal a stance favoring dual citizenship. Additionally, we find that the older the candidate is, the more likely the candidate is not to be in favor of dual citizenship. This can be considered evidence that older politicians tend to represent conservative ideology more frequently than younger politicians. Notice that most responses signaling that the candidate is against dual citizenship were sent by AfD candidates. Given that most incumbents are from the CDU/CSU and SPD, it becomes clear why the dummy for incumbency status yields a significant negative effect on the odds to receive an answer that conveys opposition to dual citizenship.

Finally, a t-test on hypothesis 3 is presented.
